# Distinct inter-hemispheric dysconnectivity in schizophrenia patients with and without auditory verbal hallucinations

**DOI:** 10.1038/srep11218

**Published:** 2015-06-08

**Authors:** Xiao Chang, Yi-Bin Xi, Long-Biao Cui, Hua-Ning Wang, Jin-Bo Sun, Yuan-Qiang Zhu, Peng Huang, Guusje Collin, Kang Liu, Min Xi, Shun Qi, Qing-Rong Tan, Dan-Min Miao, Hong Yin

**Affiliations:** 1 Department of Medical Psychology, The Fourth Military Medical University, Xi’an, Shaanxi Province, 710032, P.R. China; 2 Department of Radiology, Xijing Hospital, The Fourth Military Medical University, Xi’an, Shaanxi Province, 710032, P.R. China; 3 Department of Psychiatry, Xijing Hospital, The Fourth Military Medical University, Xi’an, Shaanxi Province, 710032, P.R. China; 4 Life Sciences Research Center, School of Life Sciences and Technology, Xidian University, Xi’an, Shaanxi Province, 710126, P.R. China; 5 Brain Center Rudolf Magnus, Department of Psychiatry, University Medical Center Utrecht, Utrecht, Netherlands

## Abstract

Evidence from behavioral, electrophysiological and diffusion-weighted imaging studies suggest that schizophrenia patients suffer from deficiencies in bilateral brain communication, and this disruption may be related to the occurrence of auditory verbal hallucinations (AVH). To increase our understanding of aberrant inter-hemispheric communication in relation to AVH, we recruited two groups of first-episode schizophrenia patients: one group with AVH (N = 18 AVH patients) and one without hallucinations (N = 18 Non-AVH patients), and 20 healthy controls. All participants received T1 structural imaging and resting-state fMRI scanning. We adopted a newly developed index, voxel-mirrored homotopic connectivity (VMHC), to quantitatively describe bilateral functional connectivity. The whole-brain VMHC measure was compared among the three groups and correlation analyses were conducted between symptomology scores and neurological measures. Our findings suggest all patients shared abnormalities in parahippocampus and striatum. Aberrant bilateral connectivity of default mode network (DMN), inferior frontal gyrus and cerebellum only showed in AVH patients, whereas aberrances in superior temporal gyrus and precentral gyrus were specific to Non-AVH patients. Meanwhile, inter-hemispheric connectivity of DMN correlated with patients’ symptomatology scores. This study corroborates that schizophrenia is characterized by inter-hemispheric dysconnectivity, and suggests the localization of such abnormalities may be crucial to whether auditory verbal hallucinations develop.

Functional segregation and functional integration are two fundamental rules of brain information processing^1^. Schizophrenia, a debilitating neuropsychiatric disorder, involves both regional brain abnormalities as well as disruptions of communication among distributed brain areas^2^,^3^ including reduced inter-hemispheric connectivity^4^,^5^,^6^. Neural activity is highly correlated between homotopic regions of the brain, especially in primary sensorimotor cortex^7^, but schizophrenia patients show reduced inter-hemispheric coherence as compared to healthy controls^8^. In addition, behavioral studies indicate impairments of lexical processing. In such studies, words and phonologically regular pseudowords are presented tachistoscopically in the left, right or bilateral visual fields. Controls respond more accurately to words presented in the bilateral condition than the unilateral conditions, which is called bilateral advantage or bilateral redundancy gain (BRG). Schizophrenia patients, however, fail to show such processing facilitation in the bilateral condition for words, indicating a deficit in inter-hemispheric cooperation^9^, that may result from neurophysiological aberrancies^10^. Furthermore, diffusion tensor imaging (DTI) studies indicate aberrances of the corpus callosum (CC), the major white-matter tract connecting the left and right cerebral hemispheres^4^,^11^. Together, these studies suggest that schizophrenia involves disrupted inter-hemispheric cooperation, due to abnormalities of inter-hemispheric structural connectivity.

Inter-hemispheric connectivity has been suggested to be particularly relevant to auditory verbal hallucinations (AVH)^12^, which are experienced by 75% of patients diagnosed with schizophrenia^13^,^14^. Prevalent theories of AVH suggest aberrancies in language related brain regions, including an hyperexcitable state of auditory cortical areas^15^ or a blurred boundary between self-generated speech and external sounds^16^. Language functions are known to be highly asymmetric, and the establishment of lateralisation has been related to normal functional segregation as well as proper communication between the hemispheres^17^,^18^. Studies suggest that functional connectivity between the hemispheres is not only involved in the establishment of functional brain asymmetry, but is also associated with psychotic symptoms^19^. Higashima *et al.* measured resting electroencephalogram (EEG) coherence in patients with schizophrenia before and after antipsychotic treatment. They found that an improvement of positive symptoms was accompanied by increased beta-band coherence of frontal electrode pairs after intervention^20^. Bleich-Cohen (2012) compared functional activation and connectivity in the left inferior frontal gyrus (IFG) among patients with schizophrenia, patients with obsessive compulsive disorder and healthy controls. They found that decreased activation asymmetry and reduced inter-hemispheric connectivity in the IFG only showed up in patients with schizophrenia, and functional connectivity between left and right IFG was significantly correlated with the lateralization index of IFG and with negative symptoms score in schizophrenia patients^21^. These results provide evidence that patients with schizophrenia display diminished language related asymmetry, and that such abnormalities are related to aberrant inter-hemispheric connectivity.

To investigate the inherent communication between two hemispheres, we employed a newly developed measurement—voxel-mirrored homotopic connectivity (VMHC), defined as the functional connectivity between each voxel in one hemisphere and its mirrored counterpart in the other hemisphere^22^. Using this index, Zuo *et al.* revealed regional variations in the lifetime development of bilateral synchronization^22^. Primary sensorimotor regions tend to show increasing connectivity between hemispheres whereas higher order regions show decreasing connectivity with age. These findings prompt interests in the physiological mechanisms underlying functional homotopy. In a range of brain disorders, including schizophrenia^23^,^24^, autism^25^, major depressive disorder^26^, mesial temporal lobe epilepsy^27^, cocaine addiction^28^ and functional dyspepsia^29^, alterations in bilateral connectivity have been identified using the VMHC index, suggesting that it is a valid indicator of inter-hemispheric connectivity changes in aging and diseases ( http://fcp-indi.github.io/docs/user/vmhc.html).

If AVH is indeed a language-related disruption, it is reasonable to speculate that inter-hemispheric connectivity may be different in schizophrenia patients suffering form AVH, as opposed to patients without AVH. In order to test this hypothesis, the current study compares inter-hemispheric functional connectivity among first-episode (FE), drug-naïve schizophrenia patients with AVH, first-episode drug-naïve patients without AVH and healthy controls. The results will be informative in answering two questions: 1. Is reduced functional integration between hemispheres a task-related phenomenon, or is it reflected in baseline neural activity? 2. Is aberrant inter-hemispheric connectivity characteristic of all patients with schizophrenia, or specific to patients with AVH? Based on previous knowledge, we predict that patients with schizophrenia will exhibit aberrant inter-hemispheric integration which differs from controls. More importantly, patients with and without AVH may demonstrate different connectivity deficits due to the involvement of inter-hemispheric connectivity in language related symptoms. This study will, for the first time, explore the origin of AVH in schizophrenia from the perspective of inter-hemispheric cooperation in resting-state fMRI.

## Methods

### Subjects

Thirty-six FE schizophrenia patients were recruited from the outpatient clinic at the Xijing Hospital, affiliated with the Fourth Military Medical University. Twenty healthy volunteers were recruited through advertisements. All participants were informed of the potential benefits and risks of this study and gave their written informed consent. The included participants were all right-handed native Chinese speakers, matched on age, gender and education level (Table 1). They were carefully examined to exclude the following conditions: a history of neurological disorder, severe medical disorders, substance abuse or dependence, prior electroconvulsive therapy or head injury resulting in loss of consciousness. This study was approved by the ethics committee of the Xijing Hospital, following the principles set forth by the Declaration of Helsinki.

Patients meeting diagnostic criteria for schizophrenia according to DSM-IV were assessed using the Positive and Negative Symptom Scale (PANSS Score ≥ 60)^30^. Two senior clinical psychiatrists performed the clinical-psychometric assessments with inter-rater reliability of more than 90%. All patients were first-episode and antipsychotic-naïve at the time of scanning. The included patients were assigned to two groups according to the presence of AVH symptom. Those who reported AVH at least once a day for the past four weeks were assigned to AVH patients group. Patients who have never experienced AVH or have not experienced them within two years before recruitment were allocated to Non-AVH group. AVH patients were further evaluated using the Auditory Hallucination Rating Scale (AHRS)^31^.

### Image acquisition

Images were acquired on a 3.0-T Siemens Magnetom Trio Tim scanner. Participants were instructed to lie still in the scanner, keeping their eyes closed but do not falling asleep. They were judged to be awake at the start and conclusion of the scanning. The participants wore a MRI-compatible head coil fitted with foam pads and earplugs to minimize head motion and dampen scanner noise. Resting-state fMRI images and high-resolution structural scan were acquired for each subject. The duration of the entire scanning process was around eight minutes.

Resting-state functional scans were acquired using a gradient-echo echo-planar imaging (EPI) sequence (TR = 2 s, TE = 30 ms, flip angle = 90^o^, FOV = 220 × 220 mm^2^). Whole-brain volumes comprised 33 contiguous transverse slices with 4 mm thickness, 0.6 mm gap and 3.40 × 3.40 mm^2^ in-plane resolution. For each participant, 240 whole-brain volumes were acquired and the first ten volumes were discarded to allow for longitudinal equilibrium. T1-weighted structural images were obtained using a three dimensional magnetization prepared rapid acquisition gradient echo (3D MPRAGE) sequence with the following parameters: TR = 2530 ms, TE = 3.5 ms, flip angle = 7^o^, FOV = 256 × 256 mm^2^. The entire brain consisted of 192 slices, with 1 mm thickness, 0.5 mm gap.

### Image Preprocessing

Data preprocessing was performed using the toolbox Data Processing Assistant for Resting-State fMRI (DPARSF)^32^ based on MATLAB (Mathworks) platform. DPARSF provides an integrated pipeline for processing resting-state fMRI data. The preprocessing section starts with slice timing and then motion correction. Data from two AVH patients were excluded from further analysis because of excessive head movement (translational >2.0 mm and/or rotational >2^o^). As recent findings suggest that “micro” head motions may also introduce group-related differences in resting-state fMRI metrics^33^,^34^,^35^, group mean framewise displacement (FD) was compared among the remaining 54 participants^36^,^37^. The FD parameters did not differ significantly among the groups (*F* = 1.09, *p* = 0.35), and were further used as covariates in subsequent group comparisons. Several sources of spurious variance, including six rigid head motion parameters, signals averaged over the lateral ventricles and deep cerebral white matter, were removed using a linear regression model. After realignment and regression, functional images were normalised to their corresponding T1 image and resampled to 3 × 3 × 3 mm^3^. The generated images were processed using spatial smoothing with an 4 mm full width at half maximum (FWHM) Gaussian kernel, linear detrend removal and temporally bandpass filtered (0.01–0.08 Hz).

### Inter-hemispheric correlation

Functional cooperation between homologous brain areas is an important feature of the brain architecture. This can be captured by resting-state connectivity between geometrically corresponding inter-hemispheric regions^7^. Zuo *et al.* put forward voxel-mirrored homotopic connectivity (VMHC), which quantifies the resting-state functional connectivity between each voxel in one hemisphere and its mirrored counterpart in the other hemisphere^22^. This index has been successfully used to quantify deficits in inter-hemispheric interaction in several brain disorders and in normal aging. In order to account for geometric differences between hemispheres, the functional images need to be registered into a symmetric template. This is achieved through a three-step processing: First, the T1 images after segmentation (e.g., wco*.img or wco*.nii) are averaged across participants to generate a mean T1 image, which is then averaged with its flipped version to generate a symmetrical T1 template; finally, the T1 image of each participant is nonlinearly registered to the symmetrical mean T1 template and this transformation is applied to their functional data. The symmetrical functional images are used to calculate Pearson correlation coefficients between all pair-wise voxels in two hemispheres, resulting in a VMHC map for every participant. Using the Fisher *r*-to-*z* transformation, the correlation coefficients are converted to *z* scores, which were used in the second-level group analyses.

### Statistic analyses

Statistic analyses were performed using REST toolbox on MATLAB platform^38^. We first conducted first-level group analyses to determine significant bilateral coherent areas (*p* < 0.01, FDR corrected, cluster size = 20). Then we performed one-way ANOVA analysis with FD parameter of head movement as covariate (*Z* = 2.3, cluster threshold at *p* < 0.05, GRF corrected), masked by a gray matter segmentation of the symmetric MNI template (thresholded at 40%)^23^. Post hoc analyses, using two-sample *t*-tests were also conducted among the three groups (*Z* = 2.3, cluster threshold at *p* < 0.05, GRF corrected). Further, we extracted neural signals from a 5-mm sphere within each aberrant region detected in the one-way ANOVA analysis. The center of sphere was set at the peak coordinate of the cluster. Averaged zVMHC values of each sphere were compared across the three groups.

### Correlation analyses

As inter-hemispheric functional interaction has been suggested to be associated with behavioral deficits in patients with schizophrenia^21^, we performed correlation analyses between PANSS total and subscale scores, and zVMHC values in all patients. In patients with AVH, we further examined relationship between ARHS score and zVMHC values. Neural signals were extracted from the 5-mm spheres of the aberrant regions.

## Results

### Group difference in VMHC

**Figure 1:**
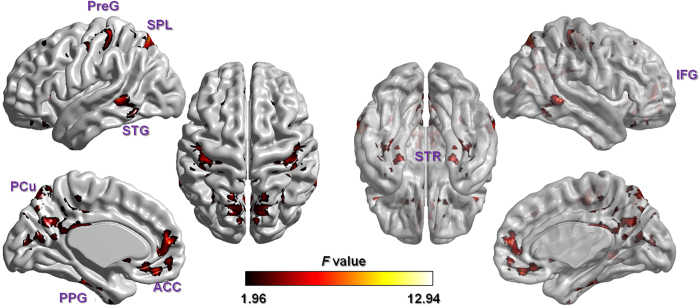
One-way ANOVA comparison on whole brain zVMHC map among three groups (Z = 2.3, cluster threshold at *p* < 0.05, GRF corrected). Results were mapped on the ICBM152 brain template using BrainNet Viewer software ( www.nitrc.org/projects/bnv/). The non-transparent left surfaces show sagittal views of the left hemisphere and an upper view of the brain. The right surface renderings show a bottom view of the brain and sagittal views of right hemisphere, with 60% opacity to show deeper structures. The cerebellum is not shown on this template. PreG: precentral gyrus; SPL: superior parietal lobule; STG: superior temporal gyrus; PCu: precuneus cortex; ACC: anterior cingulate cortex; PPG: parahippocampal gyrus; IFG: inferior frontal gyrus; STR: striatum.

Several regions showed significant differences in zVMHC values among the three groups (Fig. 1 and Table 2). These regions included language processing brain areas: posteriorof superior temporal gyrus (STG, peak coordinate: ±63, −42, 0), inferior frontal gyrus (IFG, peak coordinate: ± 53, 18, 12) anterior cingulate cortex (ACC, peak coordinate: ± 3, 54, 15), and precentral gyrus (PreG, peak coordinate: ±42, −24, 63); default mode network areas: precuneus cortex (PCu, peak coordinate: ±12, −60, 21), superior parietal lobule (SPL, peak coordinate: ±18, −69, 57) and parahippocampal gyrus (PPG, peak coordinate: ±30, −27, −30); the striatum (STR, peak coordinate: ±12, 15, 0) and the anterior lobe of the cerebellum (aCER, peak coordinate: ±18, −30, −30).

**Figure 2:**
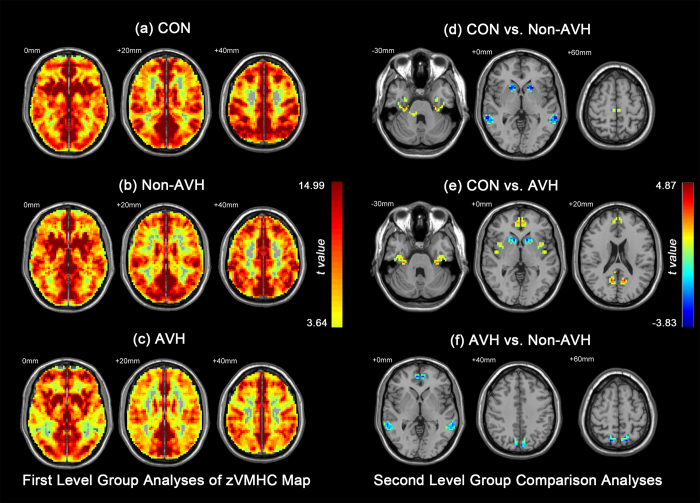
First level group analyses of zVMHC map in (**a**) control subjects; (**b**) patients without auditory verbal hallucinations (AVH); (**c**) patients with AVH. Threshold was set at *p* < 0.01, FDR corrected, cluster size = 20. The right side of the figure shows second level group comparison results between (**d**) controls vs. patients without AVH; (**e**) controls vs. patients with AVH; (**f**) patients with AVH vs. patients without AVH. Threshold was set at *Z* = 2.3 and cluster threshold at *p* < 0.05, GRF corrected. Con: control subjects; Non-AVH: patients without AVH; AVH: patients with AVH.

**Figure 3:**
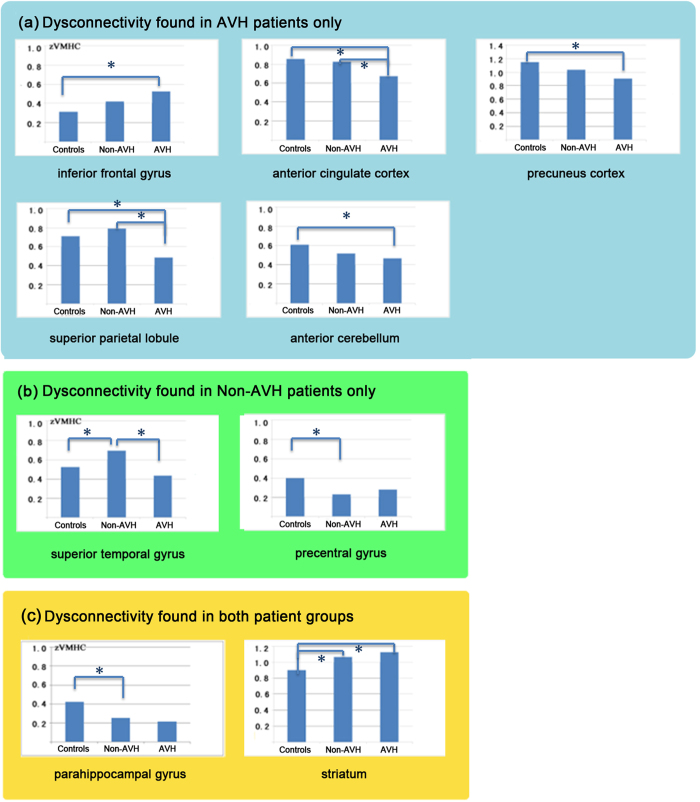
Inter-hemispheric connectivity differences between the two patient groups and healthy controls. Neural signals were extracted from a 5-mm sphere on the zVMHC map, with its center in peak coordinates of clusters showing significant group differences. (**a**) Dysconnectivity only shown in AVH Patients (**b**) Dysconnectivity only shown in Non-AVH Patients (**c**) Dysconnectivity shared by the two patient groups. Significant differences were marked with asterisks.

According to the post hoc analyses, group differences were classified into three categories: a). aberrances found in patients with AVH only, including IFG, ACC, PCu, SPL and aCER; b). aberrances in patients without AVH only, including STG and PreG; c). shared abnormalities by the two patient groups, comprising the PPG and STR (Table 3, Figs. 2,3). Significant differences in the zVMHC measure between AVH patients and Non-AVH patients were found for three regions: STG, ACC and SPL. Patients with AVH were found to show lower zVMHC values in these regions than patients without AVH, indicating that these abnormalities may relate to the different symptom profiles.

### Correlation analysis

**Figure 4:**
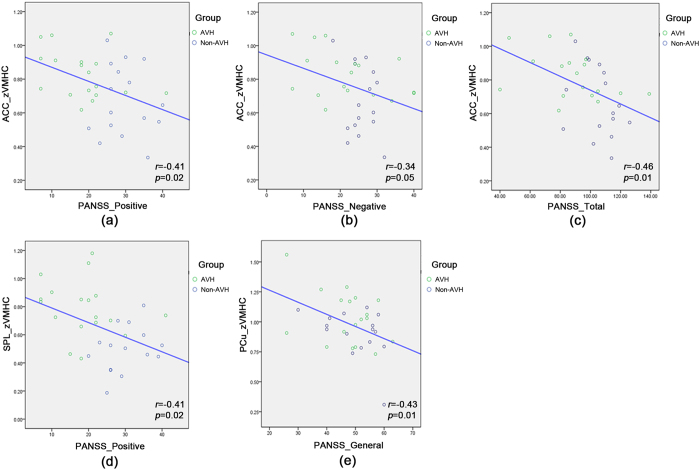
Correlation analyses between PANSS scores and zVMHC values in the two patient groups. (**a**) PANSS positive subscale score and ACC zVMHC (**b**) PANSS negative subscale score and ACC zVMHC (**c**) PANSS total score and ACC zVMHC (**d**) PANSS positive subscale score and SPL zVMHC (**e**) PANSS general psychopathology subscale score and PCu zVMHC.

*Correlations with PANSS scores in all patients –* Significant negative relationships were found between ACC zVMHC values and PANSS positive, negative, and total scores (Table 4, Fig. 4a,b,c). SPL zVMHC also showed a significant relationship with PANSS positive score (Fig. 4d). The zVMHCvalue of the PCu negatively correlated with PANSS general psychopathology score (Fig. 4e).

*Correlations with AHRS scores in AVH patients – *No significant correlations with AHRS scores were found in the AVH patients. Table S1 describes the associations between zVHMC values of each aberrant region detected in the one-way ANOVA, and AHRS scores (SI). Notably, correlations between AHRS scores and zVHMC values of two brain regions (PreG and STR) showed strong negative correlations (*r* = −0.48 and *r* = −0.49 respectively, reflecting medium-high effect size) (Figure S2), but these correlations failed to surpass trend-level (both *p* = 0.06), possibly due to the modest sample of patients with AVH (N = 16) in this study.

## Discussion

This study examined whole brain inter-hemispheric connectivity in schizophrenia patients with AVH, patients without AVH and healthy controls. Our study shows that schizophrenia is characterized by aberrant inter-hemispheric connectivity of several brain regions, including speech related brain areas, regions of the default mode network, subcortical structures and cerebellar regions. Our findings are in line with previous studies showing abnormalities of inter-hemispheric cooperation on the behavioral level, electrophysiological level and in task-based fMRI studies^9^,^10^, and extends these previous findings by showing differential abnormalities of inter-hemispheric cooperation in schizophrenia patients with AVH as compared to those without AVH.

AVH patients exhibited widespread inter-hemispheric connectivity disruptions. They showed significantly higher functional connectivity between the left IFG and its right homologous as compared to controls, with patients without AVH showing intermediate values. Functionally, the IFG (Broca’s area) is responsible for speech production. Aberrant activation of IFG in language related tasks has been reported in prior studies, which interestingly, was related to bilateral IFG connectivity, indicating that diminished language related asymmetry in the IFG was at least partly related to inter-hemispheric functional connectivity^21^. Previous research has also demonstrated the importance of the IFG in the generation of hallucinations^39^,^40^,^41^,^42^. Hoffman *et al.* examined real-time AVH experience in patients with schizophrenia or schizo-affective disorder, and found greater activation of the left IFG just before the onset of hallucinations, and stronger functional coupling between the left IFG and right temporal regions in a hallucination group compared to non-hallucinating patients^43^. In a similar experimental paradigm, Raij *et al.* found that subjective reality of AVH was correlated strongly and specifically with the IFG activation during hallucination, meanwhile, the coupling between left IFG with several other regions were also related to participants’ subjective reality of hallucination^44^. In this study, we didn’t find a significant relationship between symptomology scores and the zVMHC value of the IFG. A possible explanation is that psychotic symptoms may be more closely related to connectivity between IFG and other brain regions rather than bilateral cooperation in IFG.

Patients with AVH demonstrated abnormal bilateral connectivity of the ACC, PCu SPL and PPG, areas considered part of the default mode network (DMN). The DMN comprises a set of brain regions that maintain a high level of neural activity at wakeful rest, while they are deactivated during goal-oriented performance^45^,^46^,^47^. The DMN has been associated with internal thoughts^48^,^49^,^50^,^51^, and self-referential processing^52^,^53^. According to previous work, disruptions of DMN activity may lead to a blurred boundary between the internal and external world and improperly enhancement of self-related mental activities^3^,^54^,^55^. Such processing impairments have been suggested to be central to the generation of AVH^56^,^57^. Northoff *et al.* postulated a ‘resting state hypothesis’ of AVH, suggesting that elevated auditory cortex activity during rest and abnormal modulation by the DMN were the neuronal mechanisms underlying AVH^58^. Although still tentative, this hypothesis has been supported by many empirical and experimental studies^59^,^60^. The involvement of DMN connectivity in schizophrenia symptomology is supported by our current study showing associations between inter-hemispheric connectivity of DMN regions and PANSS total and subscale scores. Taken together, these results implicate the involvement of DMN impairments in the pathophysiological of schizophrenia symptoms.

The cerebellum has long been considered to be a brain structure that is exclusively involved in body balance and muscular coordination, but there is increasing recognition for its putative role in cognitive and affective functions and pathological involvement in schizophrenia^61^. Two previous studies also indicate decreased cerebellar VMHC in schizophrenia^23^,^24^. In the current study, we found a significant decrease in inter-hemispheric functional connectivity of the cerebellum in AVH patients and trend towards reduced cerebellar VMHC in Non-AVH patients, suggesting that inter-hemispheric dysconnection of the cerebellum may be a general deficiency in patients with schizophrenia. Notably, following the definition of Stephan *et al.* the term dysconnectivity is used to emphasize aberrant, rather than necessarily reduced, functional connectivity^3^.

Two brain regions (STG and PreG) showed potential reductions of the zVMHC index in patients without AVH, relative to AVH patients. Guo *et al.* found FE patients with paranoid schizophrenia exhibit lower VMHC in a number of regions including the STG, PCu, PreG, middle occipital gyrus (MOG), and fusiform gyrus/cerebellum. Reduced inter-hemispheric connectivity of the STG was negatively correlated with PANSS positive and negative subscale and total scores^24^, but using the same measurement, Hoptman *et al.* did not show aberrant STG zVMHC in schizophrenia patients^23^. In the current study, it is interesting to note that only Non-AVH patients showed significantly higher zVMHC values in STG, whereas AVH patients demonstrated slightly decreased values as compared to controls. A possible explanation for this result is that bilateral dysconnectivity of the STG may be a secondary impairment to primary pathological changes in AVH patients. As suggested by the corollary discharges theory (CD) of AVH, failure of communication from Broca’s area (in left IFG) to the Wernicke’s area (in left STG) may cause difficulty in distinguishing intrinsic and extrinsic sounds^42^. Abnormal bilateral connectivity in STG may be not apparent in baseline neural activity, but rather induced by hallucinations or auditive stimuli.

Decreased zVMHC value of the PreG was in line with previous studies using the same method. The studies of Hoptman *et al.* and Guo *et al.* both found significant relationships between PANSS total scores and PreG zVMHC values^23^,^24^. In the current study, we found reduced bilateral connectivity in PreG in Non-AVH patients and this aberrance correlated negatively with the PANSS positive/total scores in Non-AVH patients (*r* = −0.49/−0.51, *p* = 0.04/0.03). Meanwhile, a strong but insignificant negative correlation was found between AHRS scores and PreG zVMHC values in AVH patients (*r* = −0.48, *p* = 0.06). Reports from different modalities showing aberrant PreG connectivity, activation and morphology all hint at its crucial involvement in the pathology of schizophrenia^62^,^63^,^64^.

The two patient groups consistently showed significant alterations in STR and PPG bilateral connectivity, suggesting that these alterations may participate in the general pathological changes. The STR is a crucial component of dopaminergic pathway, and malfunction of this area may be the cause of some symptoms characteristic for schizophrenia, especially the positive symptoms^65^. We currently found trend level correlation between STR zVMHC values and AHRS scores in AVH patients (*r* = −0.49, *p* = 0.06), suggesting its putative role not only in general pathology, but also in AVH specifically. The PPG and surrounding st*r*uctures are known for their functional involvement in episodic memory processing. As abnormalities in episodic memory are the central cognitive impairment in patients with schizophrenia, the functional disruption in PPG is generally regarded to be responsible for the cognitive symptoms^66^.

There are several limitations to this study that warrant further consideration in future research. First, as a newly developed measurement, the physiological implication of VMHC index has not been clearly clarified using other imaging modalities and anatomical studies. Unlike structural connectivity that is generally decreased in patients, functional connectivity has been found to exhibit bidirectional changes, making it difficult to distinguish between primary decrease and secondary compensatory mechanisms. Earlier work of Stark *et al.* and Zuo *et al.* have shown variations in distinct brain regions and throughout the lifespan in healthy subjects, but the biological origin of VMHC and its relationship with structural connectivity remains to be explored^7^,^22^. Furthermore, before calculating VMHC, each subjects functional images were registered to a group averaged symmetric structural template, to deal with the morphologic asymmetry of the brain. This could be improved by developing more advanced algorithms to define functional homotopy (for example, clustering inter-hemispheric voxels on the basis of their resting-state connectivity) in future studies^28^.

Second, the strict criterion to recruit drug-naïve, first-episode patients limited the sample size of this study, which may have decreased our statistical power. On the other hand, this inclusion criterion has presumable minimized the influences of medication, cohort effects and illness-related environmental factors. Future studies with a larger sample size will be informative in detecting more subtle changes.

The present study indicates shared abnormalities in inter-hemispheric connectivity of STR and PPG in all patients with schizophrenia. The AVH patients exhibited significant alterations in IFG, cerebellum and several DMN regions, whereas Non-AVH patients showed differential abnormalities in STG and PreG. Moreover, inter-hemispheric functional connectivity disturbances of DMN regions negatively correlated with PANSS scores in whole patients. The current study corroborates prior findings on abnormal bilateral cooperation in patients with schizophrenia, and further extends previous knowledge by showing differential connectivity patterns in patients with and without AVH, hinting on the underlying neural mechanism of this debilitating symptom.

## Additional Information

**How to cite this article**: Chang, X. *et al.* Distinct inter-hemispheric dysconnectivity in schizophrenia patients with and without auditory verbal hallucinations. *Sci. Rep.* **5**, 11218; doi: 10.1038/srep11218 (2015).

## Supplementary Material

Supplementary Information

## Figures and Tables

**Table 1:** **Demographic and clinical characteristics of patients with AVH** **(** *n* **=** **18** **), patients without AVH** **(** *n* **= 18), and healthy controls (** *n* **= 20).**

**Characteristics**	**AVH Patients**	**Non-AVH Patients**	**Controls**
Age	22.56 ± 6.73	22.67 ± 3.85	23.43 ± 6.48
Sex (male/female)	10 / 8	9 / 9	11 / 9
Education (years)	12.44 ± 2.30	12.56 ± 2.19	13.43 ± 3.15
Duration of illness (months)	5.94 ± 5.93	12.44 ± 18.16	—
PANSS Total Score*	106.44 ± 13.55	88.06 ± 23.90	—
PANSS Positive Score*	31.11 ± 6.92	18.61 ± 8.68	—
PANSS Negative Score	25.78 ± 3.87	22.06 ± 10.43	—
PANSS General Psychopathology	49.56 ± 9.01	47.39 ± 9.84	—
AHRS Score	26.22 ± 8.10	—	—

Groups were matched for age, gender, and education

PANSS: Positive and Negative Syndrome Scale; AHRS: Auditory Hallucination Rating Scale

^*^Patients with AVH have significantly higher PANSS total and positive symptom scores than patients without AVH.

**Table 2:** **One-way ANOVA comparison on zVMHC maps among the three groups.**

**Brain areas**	**Cluster Size**	**Peak Intensity**	**Peak Coordinate (MNI)**		
			**x**	**y**	**z**
inferior frontal gyrus	103	6.79	53	18	12
anterior cingulate cortex	101	12.94	3	54	15
precuneus cortex	120	6.93	12	−60	21
superior parietal lobule	401	9.64	18	−69	57
anterior cerebellum	115	5.83	18	−30	−30
superior temporal gyrus	122	8.65	63	−42	0
precentral gyrus	174	6.51	42	−24	63
parahippocampal gyrus	232	7.99	30	−27	−30
striatum	185	8.78	12	15	0

**Table 3:** **Post hoc tests of aberrant regions in ANOVA analysis (ROI signals were extracted from a 5-mm sphere of the aberrant regions on zVMHC map).**

Brain areas	zVMHC values of ROI			*F*	Post hoc *p* values		
	Con	Non-AVH	AVH		Con vs. AVH	Con vs. Non-AVH	AVH vs. Non-AVH
*Dysconnectivity found in AVH patients only*							
inferior frontal gyrus	0.31	0.42	0.52	4.03	0.02*	0.44	0.53
anterior cingulate cortex	0.86	0.83	0.67	6.24	0.004*	1.00	0.03*
precuneus cortex	1.15	1.04	0.90	6.37	0.002*	0.31	0.18
superior parietal lobule	0.71	0.79	0.49	9.51	0.007*	0.74	0.000*
anterior cerebellum	0.61	0.52	0.45	3.22	0.05*	0.40	0.96
*Dysconnectivity found in Non-AVH patients only*							
superior temporal gyrus	0.54	0.75	0.46	7.97	0.98	0.01*	0.001*
precentral gyrus	0.40	0.23	0.28	5.03	0.12	0.01*	1.00
*Dysconnectivity found in both patient groups*							
parahippocampal gyrus	0.42	0.25	0.21	5.62	0.01*	0.04*	1.00
striatum	0.90	1.06	1.12	6.33	0.004*	0.04*	1.00

AVH: patients with auditory verbal hallucinations, Non-AVH: patients without hallucinations, Con: control subjects.

^*^Significant differences in post hoc tests are marked in bold type with an asterisk, *p* < 0.05, Bonferroni corrected.

**Table 4:** **Correlation analyses between PANSS scores and zVMHC values in all patients.**

Brain areas	PANSS Scores ( 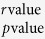 )			
	**Positive**	**Negative**	**General**	**Total**
inferior frontal gyrus	0.03	−0.02	−0.08	−0.03
	0.85	0.93	0.64	0.88
anterior cingulate cortex	**−0.41**	**−0.34**	−0.33	**−0.46**
	0.02*	0.05*	0.06	0.01*
precuneus cortex	−0.14	−0.08	**−0.43**	−0.28
	0.44	0.64	0.01*	0.11
superior parietal lobule	**−0.41**	−0.10	−0.22	−0.32
	0.02*	0.57	0.20	0.07
anterior cerebellum	−0.11	−0.07	−0.04	−0.09
	0.56	0.68	0.82	0.60
superior temporal gyrus	−0.27	−0.08	−0.15	−0.22
	0.12	0.65	0.39	0.22
precentral gyrus	0.05	0.21	0.05	0.13
	0.77	0.23	0.76	0.47
parahippocampal gyrus	−0.05	0.02	0.01	−0.01
	0.77	0.93	0.94	0.94
striatum	0.09	0.25	−0.01	0.13
	0.62	0.16	0.94	0.46

^*^Pearson correlation *p* < 0.05, significant relationship is marked in bold type.
