# Supplementary material for: Distinct inter-hemispheric dysconnectivity in schizophrenia patients with and without auditory verbal hallucinations
Source: Sci Rep. 2015 Jun 8;5:11218. doi: 10.1038/srep11218 (PMC4459220; doi:10.1038/srep11218)
Supplement: Supplementary Information [file srep11218-s1.pdf]

## Title Page

# Distinct inter-hemispheric dysconnectivity in schizophrenia patients with and without auditory verbal hallucinations

Xiao Chang*[1], Yi-Bin Xi*[2], Long-Biao Cui*[2], Hua-Ning Wang*[3], Jin-Bo Sun[4], Yuan-Qiang Zhu[4], Peng Huang[1], Guusje Collin[5,6], Kang Liu[2], Min Xi[3], Shun Qi[2], Qing-Rong Tan[3], Dan-Min Miao[#1], Hong Yin[#2]

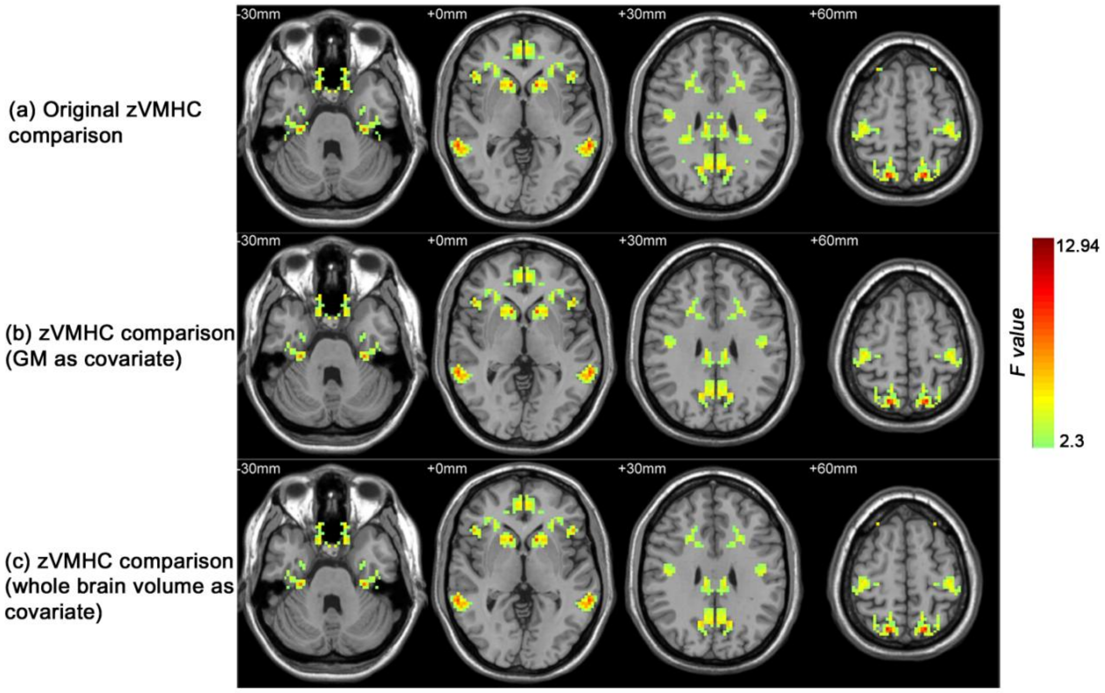


**Figure S1.** Supplementary analyses including GM and whole brain volume as covariates in zVMHC group comparisons. (a) Original one-way ANOVA result (b) Additional comparisons using total grey matter volume and (c) Whole brain volume as covariates. Statistical results were almost identical in the three conditions, implicating little impact from structural differences on zVMHC index.

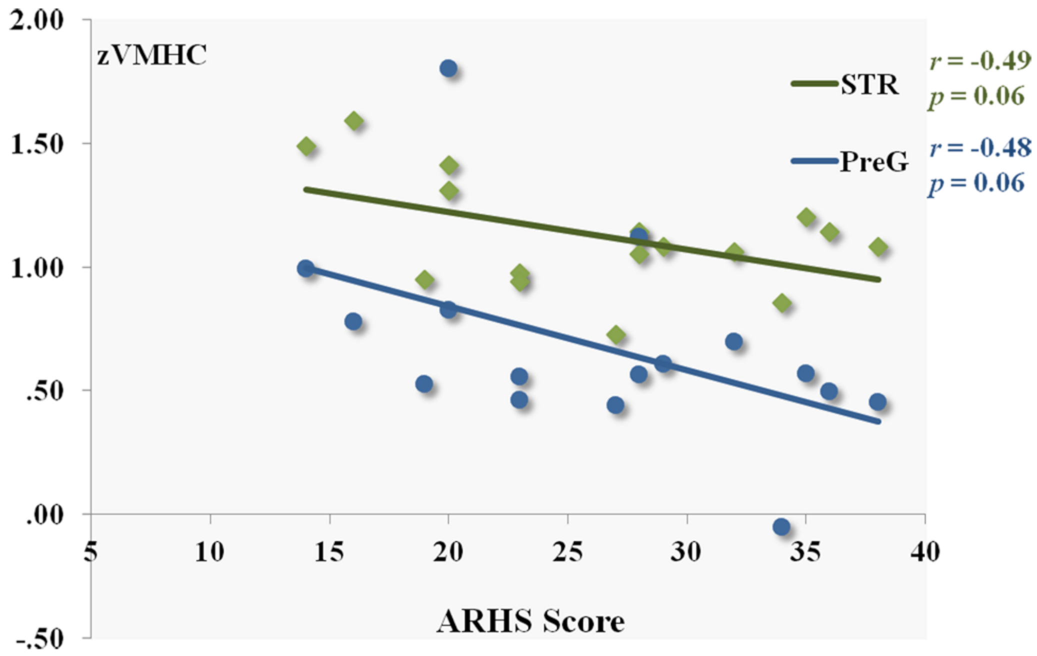


**Figure S2**. Trend level correlations between STR/PreG zVMHC measures and ARHS scores.

Table S1. Correlation between zVMHC values and ARHS scores

(Signals were extracted from a 5-mm sphere of the aberrant regions on zVMHC map)

| | | **IFG** | **ACC** | **PCU** | **SPL** | **CELLUM** | **STG** | **PreG** | **PPG** | **STR** |
|---|---|---|---|---|---|---|---|---|---|---|
| **Hoffman Score** | ***r*** | -0.36 | -0.35 | -0.19 | 0.35 | -0.03 | 0.01 | **-0.48** | 0.36 | **-0.49** |
| | ***p*** | 0.18 | 0.19 | 0.48 | 0.18 | 0.91 | 0.97 | **0.06** | 0.18 | **0.06** |

The zVMHC values in striatum (STR) and precentral gyrus (PreG) showed marginally significant correlations with AHRS scores (marked in bold type) in patients with auditory verbal hallucinations.
